# Rats maintain a binocular field centered on the horizon

**DOI:** 10.12688/f1000research.2-176.v1

**Published:** 2013-08-16

**Authors:** Markus Meister, David Cox

**Affiliations:** 1 Division of Biology, California Institute of Technology, Pasadena, CA 91125, USA; 2 Department of Molecular and Cellular Biology, Center for Brain Science, Harvard University, Cambridge, MA 02138, USA

## Abstract

In this letter, we attempt to correct a potentially serious misperception arising from the paper “Rats maintain an overhead binocular field at the expense of constant fusion”. While the authors repeatedly emphasize that the animal’s binocular field is overhead, the authors’ own data show that the truth is quite different, even orthogonal: the binocular field is in fact centered dead-ahead in front of the animal, tapering to a sliver both above *and* below the animal.  We predict that this paper will be widely cited for something that it does not demonstrate, a concern that is borne out by the paper’s earliest citation.

## Correspondence

We wish to correct a potentially serious misperception that arises from the paper "Rats maintain an overhead binocular field at the expense of constant fusion" by Wallace *et al.*, 2013 ^1^. The title, the abstract and the discussion all emphasize the principal claim that the rat's eye movements "keep the visual fields of the two eyes continuously overlapping above the animal". The final sentence reads, "Instead, the movements keep the animal’s binocular visual field above it continuously while it is moving". Many similar statements are found throughout the text and in the Editor's Summary. From this, a reader might easily conclude that a rat's binocular visual field is located overhead. However the truth is very different, and in fact orthogonal: the binocular field is primarily located dead ahead of the animal. As shown in Figure 5f of the same paper – where the vertical axis represents "overhead" in the conventional "opposite-to-gravity" sense of the word – the binocular field is a vertical sliver centered on the horizon, where it has the widest extent. It narrows towards the top and the bottom, and ends in a point at locations both above and below the animal.

Presumably the authors wanted to say that "the overhead direction is within the binocular field roughly 50% of the time", which would be more accurate given the data in Figure 5f. Of course the same is true for a direction pointing almost straight down towards the ground. Meanwhile, the direction in front of the animal is effectively always in the binocular field. Why the singular focus on "overhead"? The authors speculate about the need for binocular vision overhead for detecting predatory birds. This is unconvincing. First, only about a quarter of the overhead visual field is binocular (Figure 5f). Given the high cost of missing a predator, it seems the rat must have monocular ways of detecting one. Second, a major benefit of binocular vision is the opportunity for depth measurement by parallax. This works only for nearby objects, rather than distant birds, and would thus apply primarily to the visual field ahead or below the animal.

The authors conducted a behavioral study to reinforce the idea that rat vision is specialized for processing overhead threats, showing that rats seek shelter under an arch-shaped platform when a drifting bar appeared overhead, but not when it appeared on the side of their enclosure (Figure 6). While we hesitate to engage in behavioral just-so stories, we do note that different sheltering strategies probably exist for different kinds of threats (e.g. land-based and aerial), and that the rat might be wise not enter into a confined space *with open sides*, when a threat approaches *from the side*. It is likewise unclear if a drifting bar is equally perceived as a threat when presented above or beside the animal.

Regardless of such ethological speculations, we predict that the paper will be widely cited incorrectly for what it does not demonstrate, as a result of the misleading title and abstract. In fact, this has happened already in what is probably the article's first citation ^2^.
