# Supplementary material for: Rats maintain a binocular field centered on the horizon
Source: F1000Res. 2013 Aug 16;2:176. [Version 1] doi: 10.12688/f1000research.2-176.v1 (PMC3790602; doi:10.12688/f1000research.2-176.v1)
Supplement: Peer review history

# Peer review: Referee response for version 1

**Published:** 2013-08-28

**Authors:** Michael Land

**Affiliations:** 1 University of Sussex, Brighton, UK

I approve of the authors' commentary. The mistake in the original paper comes from a confusion where the binocular field is relative to the head, rather than where it is relative to gravity - which is actually the relevant measurement. Cox and Meister are right to point this out. It is certainly true that the field moves as the head moves, because of the compensatory eye movements, but it remains only partially overhead, and has its maximal extent in the forward direction.

I have read this submission. I believe that I have an appropriate level of expertise to confirm that it is of an acceptable scientific standard.

# Peer review: Referee response for version 1

**Published:** 2013-08-22

**Authors:** Stephen Van Hooser

**Affiliations:** 1 Brandeis University, Waltham, MA, USA

The present letter refers to a recent (2013) Nature article by Wallace, Greenberg, Sawinski, and colleagues entitled 'Rats maintain an overhead binocular field at the expense of constant fusion'. This Nature article is a tour-de-force examination of eye movements in freely moving rats. The authors created instrumentation and analysis methods to both examine eye movements and rotations, and head movements and rotations, while the rats behaved freely. They found the surprising result that the eyes do not maintain constant fusion, as they do in previously examined animals such as primates. Further, from their analysis of eye movements and head movements, the authors determined the most common region of binocular overlap as the animals wandered about their environment. In head-centric coordinates, this region of binocular overlap reached its greatest horizontal width at a location superior and rostral to the nose (Figure 5e). The authors work also shows that rats tend to keep their heads pitched downward as they move about. Therefore, in body-centric coordinates (using the spine to determine rostral, caudal, superior, inferior directions), this region of binocular overlap reaches its greatest horizontal width at a location directly rostral to the body (Figure 5f).

The present letter by Meister and Cox raises a legitimate and serious concern with the "big picture" language of the Wallace *et al*. paper. In the abstract, Wallace *el al.* report that "the observed eye movements serve to keep the visual fields of the two eyes continuously overlapping above the animal during free movement, but not continuously aligned". The coordinate system for the word "above" is not clear from context.  Meister and Cox makes the important point that a substantial fraction of readers (perhaps an overwhelming majority) are likely to assume that "above" is in body-centric coordinates, and to make the erroneous interpretation that the binocular overlap is largest above the animal. That is, many readers will erroneously interpret this sentence to mean that the region of greatest binocular overlap is superior to the spine. This potential misinterpretation is made even more likely by the behavioral experiment reported in Wallace *et al*., where the authors provide visual stimulation above the spine. The letter writers also point out that there is no rigorous link between the behavioral experiment and the region of binocular overlap. While the behavioral result is interesting, it is unclear if the behavior has anything to do with binocular vision.

This letter by Meister and Cox makes a very important contribution. In my opinion, the letter could be improved by making clearer references to the coordinate system being used when a direction is mentioned. For example, the letter writers state "the binocular visual field is located dead ahead of the animal". The writers assume that the reader will intuit that body-centric coordinates are intended. However, some readers (such as the authors of Wallace *et al*.) may find it more natural to assume that head-centric coordinates are intended. I understand what the authors mean by "opposite-to-gravity"; the authors assume the animal is on its 4 legs. Referring to coordinate systems linked to the animal would be clearer, in my opinion.

I have read this submission. I believe that I have an appropriate level of expertise to confirm that it is of an acceptable scientific standard.

# Peer review: Referee response for version 1

**Published:** 2013-08-19

**Authors:** Matteo Carandini

**Affiliations:** 1 Institute of Ophthalmology, University College London, London, UK

This commentary can perhaps be reconciled with the original paper by considering the meaning of 'overhead'. In the original paper, 'overhead' seems to mean literally 'above the head'. Because the head typically faces downward (compare Figures 5e and 5f), that actually means 'in front of the animal'. I agree with the commentary that this is possibly a poor word choice: it will likely be interpreted by most readers as 'above the animal'.

I have read this submission. I believe that I have an appropriate level of expertise to confirm that it is of an acceptable scientific standard.
